# Supplementary material for: Pyruvate Dehydrogenase Kinase 1 inhibition mediated oxidative phosphorylation enhancement in cartilage promotes osteoarthritis progression
Source: BMC Musculoskelet Disord. 2023 Jul 20;24:597. doi: 10.1186/s12891-023-06585-6 (PMC10357736; doi:10.1186/s12891-023-06585-6)
Supplement: Supplementary file 1 — Additional file 1. [file 12891_2023_6585_MOESM1_ESM.zip › BP.Gsea.1653623667859/GOBP_POSITIVE_REGULATION_OF_FATTY_ACID_METABOLIC_PROCESS.html]

Details for gene set GOBP\_POSITIVE\_REGULATION\_OF\_FATTY\_ACID\_METABOLIC\_PROCESS[GSEA]

|  || Dataset | 55457\_collapsed\_to\_symbols.cls.cls #OA\_versus\_normal.cls.cls #OA\_versus\_normal\_repos |
| Phenotype | cls.cls#OA\_versus\_normal\_repos |
| Upregulated in class | normal |
| GeneSet | GOBP\_POSITIVE\_REGULATION\_OF\_FATTY\_ACID\_METABOLIC\_PROCESS |
| Enrichment Score (ES) | -0.51157653 |
| Normalized Enrichment Score (NES) | -1.7636087 |
| Nominal p-value | 0.0033167496 |
| FDR q-value | 0.11409789 |
| FWER p-Value | 0.998 |
Table: GSEA Results Summary

  

Fig 1: Enrichment plot: GOBP\_POSITIVE\_REGULATION\_OF\_FATTY\_ACID\_METABOLIC\_PROCESS      
 Profile of the Running ES Score & Positions of GeneSet Members on the Rank Ordered List

  

| SYMBOL | TITLE | RANK IN GENE LIST | RANK METRIC SCORE | RUNNING ES | CORE ENRICHMENT || 1 | RGN | regucalcin [Source:HGNC Symbol;Acc:HGNC:9989] | 1369 | 0.448 | -0.0686 | No |
| 2 | NR1H3 | nuclear receptor subfamily 1 group H member 3 [Source:HGNC Symbol;Acc:HGNC:7966] | 1728 | 0.394 | -0.0632 | No |
| 3 | APOC2 | apolipoprotein C2 [Source:HGNC Symbol;Acc:HGNC:609] | 1855 | 0.379 | -0.0411 | No |
| 4 | ABCD2 | ATP binding cassette subfamily D member 2 [Source:HGNC Symbol;Acc:HGNC:66] | 3031 | 0.246 | -0.1117 | No |
| 5 | MID1IP1 | MID1 interacting protein 1 [Source:HGNC Symbol;Acc:HGNC:20715] | 3559 | 0.197 | -0.1360 | No |
| 6 | PLA2G3 | phospholipase A2 group III [Source:HGNC Symbol;Acc:HGNC:17934] | 3648 | 0.190 | -0.1268 | No |
| 7 | MLYCD | malonyl-CoA decarboxylase [Source:HGNC Symbol;Acc:HGNC:7150] | 4852 | 0.083 | -0.2132 | No |
| 8 | NR1H2 | nuclear receptor subfamily 1 group H member 2 [Source:HGNC Symbol;Acc:HGNC:7965] | 5413 | 0.039 | -0.2535 | No |
| 9 | PPARG | peroxisome proliferator activated receptor gamma [Source:HGNC Symbol;Acc:HGNC:9236] | 6006 | -0.005 | -0.2991 | No |
| 10 | CD74 | CD74 molecule [Source:HGNC Symbol;Acc:HGNC:1697] | 6374 | -0.033 | -0.3248 | No |
| 11 | LPGAT1 | lysophosphatidylglycerol acyltransferase 1 [Source:HGNC Symbol;Acc:HGNC:28985] | 6519 | -0.043 | -0.3323 | No |
| 12 | APOA4 | apolipoprotein A4 [Source:HGNC Symbol;Acc:HGNC:602] | 6817 | -0.064 | -0.3499 | No |
| 13 | AVP | arginine vasopressin [Source:HGNC Symbol;Acc:HGNC:894] | 7201 | -0.096 | -0.3716 | No |
| 14 | FABP1 | fatty acid binding protein 1 [Source:HGNC Symbol;Acc:HGNC:3555] | 7604 | -0.127 | -0.3921 | No |
| 15 | ADIPOQ | "adiponectin, C1Q and collagen domain containing [Source:HGNC Symbol;Acc:HGNC:13633]" | 7792 | -0.141 | -0.3948 | No |
| 16 | CPT1A | carnitine palmitoyltransferase 1A [Source:HGNC Symbol;Acc:HGNC:2328] | 8046 | -0.159 | -0.4011 | No |
| 17 | GHSR | growth hormone secretagogue receptor [Source:HGNC Symbol;Acc:HGNC:4267] | 8472 | -0.191 | -0.4180 | No |
| 18 | APOA1 | apolipoprotein A1 [Source:HGNC Symbol;Acc:HGNC:600] | 8882 | -0.223 | -0.4309 | No |
| 19 | ABCD1 | ATP binding cassette subfamily D member 1 [Source:HGNC Symbol;Acc:HGNC:61] | 8930 | -0.226 | -0.4155 | No |
| 20 | MLXIPL | MLX interacting protein like [Source:HGNC Symbol;Acc:HGNC:12744] | 9882 | -0.310 | -0.4633 | No |
| 21 | PPARGC1A | PPARG coactivator 1 alpha [Source:HGNC Symbol;Acc:HGNC:9237] | 10504 | -0.374 | -0.4801 | Yes |
| 22 | ANXA1 | annexin A1 [Source:HGNC Symbol;Acc:HGNC:533] | 10824 | -0.407 | -0.4705 | Yes |
| 23 | PPARA | peroxisome proliferator activated receptor alpha [Source:HGNC Symbol;Acc:HGNC:9232] | 11110 | -0.445 | -0.4552 | Yes |
| 24 | ELOVL5 | ELOVL fatty acid elongase 5 [Source:HGNC Symbol;Acc:HGNC:21308] | 11272 | -0.467 | -0.4283 | Yes |
| 25 | PPARD | peroxisome proliferator activated receptor delta [Source:HGNC Symbol;Acc:HGNC:9235] | 11301 | -0.471 | -0.3907 | Yes |
| 26 | IRS2 | insulin receptor substrate 2 [Source:HGNC Symbol;Acc:HGNC:6126] | 11390 | -0.485 | -0.3567 | Yes |
| 27 | TWIST1 | twist family bHLH transcription factor 1 [Source:HGNC Symbol;Acc:HGNC:12428] | 11696 | -0.531 | -0.3356 | Yes |
| 28 | IRS1 | insulin receptor substrate 1 [Source:HGNC Symbol;Acc:HGNC:6125] | 11841 | -0.557 | -0.2998 | Yes |
| 29 | IL1B | interleukin 1 beta [Source:HGNC Symbol;Acc:HGNC:5992] | 12105 | -0.622 | -0.2678 | Yes |
| 30 | NR4A3 | nuclear receptor subfamily 4 group A member 3 [Source:HGNC Symbol;Acc:HGNC:7982] | 12543 | -0.769 | -0.2369 | Yes |
| 31 | PTGS2 | prostaglandin-endoperoxide synthase 2 [Source:HGNC Symbol;Acc:HGNC:9605] | 12641 | -0.820 | -0.1753 | Yes |
| 32 | AVPR1A | arginine vasopressin receptor 1A [Source:HGNC Symbol;Acc:HGNC:895] | 12751 | -0.913 | -0.1068 | Yes |
| 33 | AKT2 | AKT serine/threonine kinase 2 [Source:HGNC Symbol;Acc:HGNC:392] | 12896 | -1.404 | 0.0003 | Yes |
Table: GSEA details [plain text format]

  

Fig 2: GOBP\_POSITIVE\_REGULATION\_OF\_FATTY\_ACID\_METABOLIC\_PROCESS      
 Blue-Pink O' Gram in the Space of the Analyzed GeneSet

  

Fig 3: GOBP\_POSITIVE\_REGULATION\_OF\_FATTY\_ACID\_METABOLIC\_PROCESS: Random ES distribution      
 Gene set null distribution of ES for **GOBP\_POSITIVE\_REGULATION\_OF\_FATTY\_ACID\_METABOLIC\_PROCESS**
